# ﻿*Adesmiaephedroides* (Fabaceae, Faboideae), a new species from the Mediterranean-type ecosystem of Valparaíso Region, Chile

**DOI:** 10.3897/phytokeys.259.156135

**Published:** 2025-07-10

**Authors:** Benito Rosende, Nicolás Lavandero, Daniela Araneda, María Fernanda Pérez

**Affiliations:** 1 Facultad de Ciencias Biológicas, P. Universidad Católica de Chile, Santaigo, Chile P. Universidad Católica de Chile Santiago Chile

**Keywords:** Central Chile, Dalbergieae, endemism, Leguminosae, matorral, new species, Papilionoideae, rupicolous flora, taxonomy

## Abstract

A new species, *Adesmiaephedroides*, discovered in a restricted area of the coastal mountain range within the Valparaíso Region, Chile, is here described. Molecular phylogenetic analyses, based on five nuclear loci, were carried out to explore the phylogenetic position of the new species. Our findings robustly support *A.ephedroides* as a distinct species, positioned within a clade of *Adesmia*, characterised by shrubby growth habit, racemose inflorescences and bristled lomenta. This identified clade exhibits a distribution limited to the semi-arid regions spanning from the southern Atacama Desert to Central Chile. *Adesmiaephedroides* is readily distinguishable from its closest relatives through a combination of features, including green young branches and distinctive leaf morphology: glabrous, few leaflets and persistent, cylindrical petiole and rachis. A detailed taxonomic description, illustrations, field images, distribution map, ecological insights into its habitat and a preliminary IUCN conservation status assessment are provided.

## ﻿Introduction

*Adesmia* DC. (Fabaceae, Faboideae) belongs to the tribe Dalbergieae*sensu lato* (Lavin et al. 2001; Choi et al. 2022), where the monogeneric clade Adesmieae is characterised by shrubs, subshrubs, annual and perennial herbs, exhibiting free stamen filaments and lomentaceous pods (Polhill 1981).

Endemic to South America, the genus Adesmia is distributed across Peru, Bolivia, Chile, Argentina, Uruguay and Brazil. This distribution reflects the genus’ capacity to colonise arid and/or cold climates (Iganci and Miotto 2011; Fortuna-Perez et al. 2021), aligning with the western South American Arid Diagonal (wSADD) (Luebert 2021) from the Peruvian Andes to Patagonia, with extensions eastwards into eastern Argentina to the cold uplands of southern Brazil (Burkart 1967).

*Adesmia* DC. encompasses between 206 and 230 species (Burkart 1967; POWO 2025), making it one of the most species-rich genera within Fabaceae in southern South America (Burkart 1967; Monteiro et al. 2024). The most comprehensive infrageneric classification, proposed by Burkart (1967), divides the genus into two subgenera, *Adesmia* and *Acanthadesmia*, based on the absence or presence of spines, respectively. Furthermore, he proposed 43 series, relying on morphological traits such as leaf morphology, inflorescences and fruit indumentum (Burkart 1967). However, recent phylogenetic studies indicate that the presence/absence of spines or plumose trichomes in fruits is not a strong indicator of clade relationships within the genus (Monteiro et al. 2024; Pérez et al. 2024). Two primary centres of diversification for *Adesmia* have been identified: a semi-arid Andean region between the southern Atacama Desert and central Chile and central and southern Argentina (Burkart 1967). More recent phylogenetic evidence suggests the Atacama Desert as the most likely origin centre of the genus, originating from a herbaceous ancestor (Pérez et al. 2024).

Chile harbours a significant portion of *Adesmia*’s diversity, with 130 species (56–63% of the total) and six varieties, exhibiting high levels of endemism (63%). These are distributed across a broad latitudinal (~17.5°–52.3°S) and altitudinal range (0–4300 m) (Rodríguez et al. 2018). Notably, the highest species richness is concentrated between the Atacama Desert and the central Andes of Chile, with a complete absence in evergreen temperate rainforests (Burkart 1967; Pérez et al. 2024). Central Chile is recognised as a global biodiversity hotspot (Myers et al. 2000; Arroyo et al. 2006), characterised by strong biogeographic isolation from the rest of the continent (Arroyo et al. 2006) and complex geographical heterogeneity due to two parallel mountain ranges traversing the country from north to south (Mardones and Scherson 2023). Additionally, Quaternary climatic processes likely played a crucial role in promoting speciation and high rates of endemism in this region (Arroyo et al. 1995; Villagrán 1995).

This study formally describes a new species of *Adesmia*, including illustrations, field images, detailed information on its distribution and habitat in central Chile, ecological and phenological insights and a preliminary conservation status assessment following IUCN guidelines. Furthermore, we investigated its phylogenetic relationships using molecular data to re-evaluate the phylogeny of *Adesmia* as proposed by Pérez et al. (2024). Finally, a dichotomous key is provided to distinguish the new species from other shrubby *Adesmia* taxa in the Valparaíso Region’s Coastal Range, north of the Aconcagua River.

## ﻿Methods

### ﻿Herbarium and fieldwork

During fieldwork in March 2024 at the base of Cerro Caqui (Catemu, Valparaíso Region, Chile), specimens of *Adesmia* that could not be readily assigned to any currently accepted species were discovered. A subsequent fieldwork expedition was conducted at the same locality in November 2024 to observe plants during their suspected flowering period. Specimen samples were collected, leaf material was preserved in silica gel and flowers were preserved in 70% ethanol. These specimen samples were deposited in the Herbaria SGO, CONC and EIF. The primary taxonomic literature on *Adesmia* (Burkart 1967; Ulibarri 1986) was consulted for morphological descriptions of previously described species. A systematic examination was undertaken of *Adesmia* herbarium specimens housed at CONC and SGO and of digital images available online from E, K, P, ULS and CONC. The descriptions and taxonomic keys were prepared following examination of all accessible specimens. Terminology for describing floral parts follows Simpson (2010) and Beentje (2016). Measurements of structures smaller than 1 cm were taken using a Nikon SMZ 745T stereomicroscope and photographed with a Canon EOS REBEL T3 digital camera equipped with an adapter. Larger structures were measured with the naked eye or photographed with the same camera. All width measurements represent the widest dimension of the structure and measurements of small structures were obtained using the open-source software ImageJ (Schneider et al. 2012).

### ﻿Conservation status

Following the International Union for Conservation of Nature (IUCN 2012) Categories and Criteria and following the most recent guidelines provided by the IUCN Standards and Petitions Committee (2024), a preliminary conservation status assessment was conducted for the new species. The extent of occurrence (EOO) and area of occupancy (AOO) were calculated using GeoCat (Bachman et al. 2011). Threats to the species were identified through field observations.

### ﻿Taxon sampling and phylogenetic analysis

DNA sequences of *Adesmia* from Pérez et al. (2024) were obtained from GenBank (www.ncbi.nlm.nih.gov/Genbank). This dataset consists of five loci. Three nuclear single-copy nuclear genes, the Auxin-independent growth (AIGP) gene (Choi et al. 2004), the U5 small nuclear ribonucleoprotein component CLO gene, the vacuolar-sorting receptor 1 gene, plus two regions of the nuclear ribosomal DNA (nrDNA) cistron, the internal transcribed spacer (ITS) and the external transcribed spacer (ETS). As outgroups, we included *Amiciaandicola*, *Amicialobbiana*, *Amiciamicrantha*, *Nissoliabracteosa*, *Nissoliaschottii*, *Poiretialongipes*, *Poiretiamarginata*, *Zorniaharmsiana*, *Zorniaglochidiata* and *Zorniaaerolata*. *Adesmiacapitellata* was excluded from our analyses due to the poor quality of the obtained sequences and issues in the posterior alignment. Additionally, we sampled 13 species of *Adesmia* (including the putative new species) and one variety (Adesmiapapposavar.radicifolia) that were not considered in Pérez et al. (2024).

Total genomic DNA was extracted either from silica-dried material collected in the field or from herbarium material (SGO and CONC) using the Qiagen DNeasy Plant Mini Kit (QIAGEN, Santiago, Chile) following the manufacturer’s instructions. Genomic DNA was used to amplify by PCR each of the five regions from Pérez et al. (2024), using the same primer pairs and methods specified by the authors. Sanger sequencing was performed in the Plataformas UC de Secuenciación y Tecnologías Ómicas, Pontificia Universidad Católica de Chile, using the ABI PRISM 3500 xl Genetic Analyzer (Applied Biosystems™). GenBank accession numbers for all DNA sequences used in this study are given in Suppl. material 1.

The assembled sequences were aligned using the MAFFT v.7.450 algorithm (Katoh et al. 2002; Katoh and Standley 2013) in Geneious Prime 2022.2.1 (https://www.geneious.com). Phylogenetic analyses were run for both Maximum Likelihood (ML) (Felsenstein 1981), using RAxML-AVX3 version (Stamatakis 2014) included in RAxMLGUI v.2.0 beta (Silvestro and Michalak 2012; Edler et al. 2020) and Bayesian Inference (BI) using MrBayes x64 v3.2.7 (Ronquist et al. 2012), respectively. The best-supported model of nucleotide sequence evolution for each region was determined, based on the Akaike Information Criterion (AIC) using MrModelTest v.2 (Nylander 2004). For the vacuolar-sorting receptor 1 gene and the ITS regions, the GTR+I+G model was selected. For the AIGP gene, U5 small nuclear ribonucleoprotein component CLO gene and the ETS region, the HKY+G model was selected. Bayesian analyses were conducted under the respective best-fit models for each partition, with two independent runs for 20 million generations, sampling every 10,000 generations. Time series plots and effective sample size (ESS) were analysed using TRACER v.1.7 (Rambaut et al. 2018) to check convergence for each run. The first 4 million generations were discarded as burn-in. Maximum Likelihood analyses were run using the GTRGAMMA approximation, considering gene partitions and including the proportion of invariant sites (+I option). The analysis included 1000 ML slow bootstrap replicates with 100 runs.

## ﻿Results

### ﻿Molecular phylogenetic analyses

The total DNA alignment contained 2860 characters (673 ITS, 336 ETS, 349 AIGP, 710 vacuolar-sorting receptor 1 gene, 792 U5 small nuclear ribonucleoprotein component CLO gene), representing 93 ingroup and 10 outgroup accessions. The tree topology obtained by BI and ML is similar to the one obtained by Pérez et al. (2024), although there are several noteworthy differences (Fig. 2). In the ML tree, as sister to all *Adesmia* taxa, a clade comprised by *Adesmiafuentesii*, *Adesmiajilesiana* and *Adesmiamulticuspis* is found. In BI analyses, *Adesmiafuentesii* and *Adesmiajilesiana* form a clade that is sister to a clade formed only by *Adesmiamulticuspis* and the remaining species. In Pérez et al. (2024), *Adesmiamulticuspis* appears as sister to all *Adesmia* species, followed by a clade formed by *Adesmiafuentesii* and *Adesmiajilesiana* (Clade A). Clade B1 (PP = 1.0; BS = 100) and B2 (PP = 1.0; BS = 99) *sensu*Pérez et al. (2024) are consistently retrieved by both analyses, although *Adesmialongipes*’ position in BI is unresolved. Clade B2 includes the newlysequenced Adesmiapapposavar.radicifolia and *Adesmiacodonocalyx*. Clade C *sensu*Pérez et al. (2024) is consistently retrieved (PP = 1.0; BS = 71), also including the newly-sampled *Adesmiabalsamica*. Clade D is consistently retrieved with high support (PP = 1.0; BS = 100), including the newly-sampled *Adesmiasalicornioides*. Lastly, *Adesmiacorymbosa* is retrieved by both analyses as sister to clade E. Clade E1 differs from Pérez et al. (2024) in the ML tree, as it is not resolved as a sister clade to E2 as in the BI tree. BI includes in E1 (PP = 0.65) the newly-sequenced *Adesmiarubroviridis*, *Adesmiamonosperma*, *Adesmiaobscura*, *Adesmiatrifoliata*, *Adesmiaocculta* and *Adesmiasubterranea*. Clade E2 is retrieved with high support (PP = 1.0; BS = 94), including the newly-sampled *Adesmiaargentea* and the putative new species, *Adesmiaephedroides*. ML analyses retrieve E2 as sister to a clade formed by *Adesmiaobscura* and *Adesmiamonosperma* and includes *Adesmiahystrix*, all belonging to Clade E1 in BI analyses. Clade E3, including *Adesmiaatacamensis* and *Adesmiaverrucosa*, is retrieved with high support in BI (PP = 1.0) and moderately supported in ML (BS = 53).

### ﻿Taxonomic treatment

#### ﻿ Adesmia ephedroides

Taxon classificationPlantaeFabalesFabaceae

Rosende & Lavandero sp. nov.

0BB67CD1-566B-5FCD-AF16-E05EF9A8FCC7

urn:lsid:ipni.org:names:77365253-1

Figs 3 , 4 , 5

##### Diagnosis.

*Adesmiaephedroides* differs from its closest relatives by the following combination of characters: unarmed shrubs with glabrous, green young branches; uniquely-shaped leaves with a persistent, cylindrical petiole and rachis; 1–3 pairs of glabrous, obovate to oblong, deciduous leaflets with an emarginate apex and a margin dentate with glandular setae and 1–3 articles, plumose bristled lomenta.

##### Type.

Chile • [1 ♀♂, 1.3 m]; Región de Valparaíso, Provincia de San Felipe de Aconcagua, Comuna de Catemu, Southwest of Cerro Caqui; 32°45'45.1"S, 70°59'52.2"W; 709 m alt.; 2 November 2024; *B. Rosende & N. Lavandero* leg.; fl, fr.; (holotype: SGO [SGO171977]!; Isotype: SGO [SGO171978]!.).

##### Description.

***Shrubs*** (0.2) 0.4–1.6 m tall and 0.3–0.8 m width; ***Stems*** woody, cylindrical, branched at the base, bifurcating, greyish bark longitudinally fissured; branches oblique to vertically orientated, nearly terete, glaucous, green to reddish-brown with age, glabrous, internodes (7)12–22 mm long, short or hardly visible brachyblast on axillary nodes; ***Stipules*** triangular-ovate, 0.5–0.7 mm long, base 0.6 to 0.8 mm wide, glabrescent, green to pink, persistent; ***Leaves*** alternate, paripinnate, rarely imparipinnate (trifoliolate, at the leaf apex), 1–3 pairs of opposite leaflets, glabrous with few ciliate trichomes at the petiole base, leaflets pairs widely spaced and orientated to the leaf apex; ***Petiole*** 19.5–55 mm long; ***Rachis*** 1–18 mm long, cylindrical, canaliculated when dry, persistent; ***Leaflets*** 0.5–2.8 width × 0.6–2.3 mm long, subsessile, obovate or oblong, apex emarginate or rounded, base cuneated, margin entire or irregularly dentate towards the apex with few setulae with a glandular base, located at the tips of teeth, glabrescent; leaf blade spreading, subconduplicate or concave; deciduous; ***Raceme*** terminal occasionally axillary, (1.7)7–15 cm long, erect, glabrous, 5–27 flowered; ***bracts*** 0.5–1.5 mm long, triangular-lanceolate, glabrous; ***pedicel*** 5–13.5 mm long, spread or erect, glabrous; ***Flowers*** yellow, hermaphrodite, 5.9–8.3 mm long; **c*alyx*** campanulate, 3.3 × 2.5 mm, externally glabrous, internally pubescent, margin ciliate, 5-lobed, 0.3–7 mm long, dorsal sinus narrower and deeper than the other four; ***standard petal*** blade yellow with dark red nectar guides, redder externally, reflexed, orbicular to broadly obovate, apex emarginate, 5.3–6.2 × 3.9 mm, externally pubescent on the veins, claw white, internally pubescent distally,1.9–2.1 mm long; ***wing petals*** glabrous, falcate, blade yellow, 13.6–18.3 × 8.4–9.9 mm, narrowly obovate, apex obtuse, curved inwards, with a spur ca. 1.8 mm long, claw white 10.6–12.9 mm long; ***keel petals*** falcate, blade 14.7–15.2 × 8.7–9.5 with a spur ca. 0.7 mm long, ciliate along the lower margin, apex acute, claw 10.3–11.7 mm long; ***stamens*** 10, filaments free, flattened, curved upwards, 2 stamens filaments are thicker than the others, 18.3–28.4 mm long, glabrous; ***anthers*** elliptical, dorsifixed; ***gynoecium*** 6.6–8.4 mm long; ***ovary*** straight 3.7–8.4 mm long, pubescent, 1–3 ovules; ***style*** curved upwards, 3.3–4.3 mm long; ***stigma*** punctiform; ***Fruit*** lomentum, not stipitate, calyx persistent, sometimes embracing the first article of the fruit; laterally compressed, articles 1–3, semi-lenticular, 6.0–6.4 × 7–8.1 mm, each article with dark red bristles with numerous spread white hairs, bristles bended distally, 18.6–30.4 mm; ***Seed*** brownish, 2 × 1.8 mm, orbicular to lenticular; hilum orbicular, no aril.

##### Distribution and habitat.

**Figure 1.**
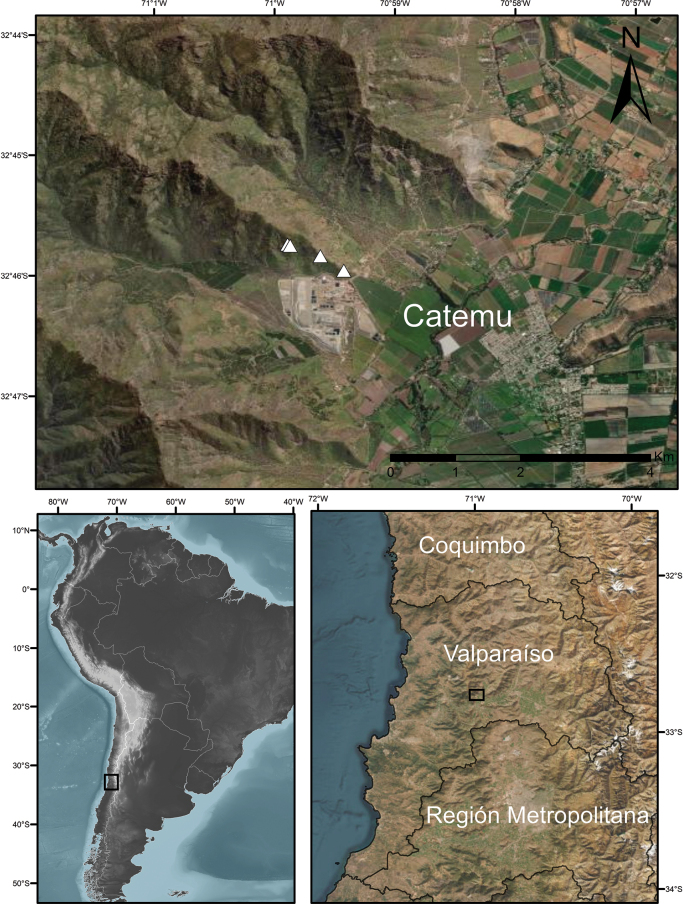
Distribution map of *Adesmiaephedroides* (white triangles) in Chile, Valparaíso Region, based on the type locality and collections by the authors (paratypes).

*Adesmiaephedroides* is endemic to the Valparaíso Region, currently known only from the type locality in the Melón Mountain Range (coastal central Chile; Fig. 1). It inhabits crevices of south- to southeast-facing rocky outcrops between 530 and 720 m alt. The surrounding sclerophyllous open forest/shrubland is dominated by the tree species *Quillajasaponaria* Poir. (Quillajaceae) and *Lithraeacaustica* (Molina) Hook. & Arn. (Anacardiaceae), with *Retanillatrinervia* Hook. & Arn. (Rhamnaceae), *Porlieriachilensis* I.M.Johnst. (Zygophyllaceae) and *Colliguajaodorifera* Molina (Euphorbiaceae) as common shrubs. Associated rock outcrop species include Calceolariaascendenssubsp.exigua (Witasek) Nic.García (Calceolariaceae), *Stachys* sp. L. (Lamiaceae), *Ephedrachilensis* Miers (Ephedraceae), *Lobeliaexcelsa* Lesch. (Campanulaceae), *Leucostelechiloensis* (Colla) Schlumpb. (Cactaceae) and Puyaalpestrissubsp.zoellneri Zizka, J.V.Schneid. & Novoa (Bromeliaceae).

**Figure 2.**
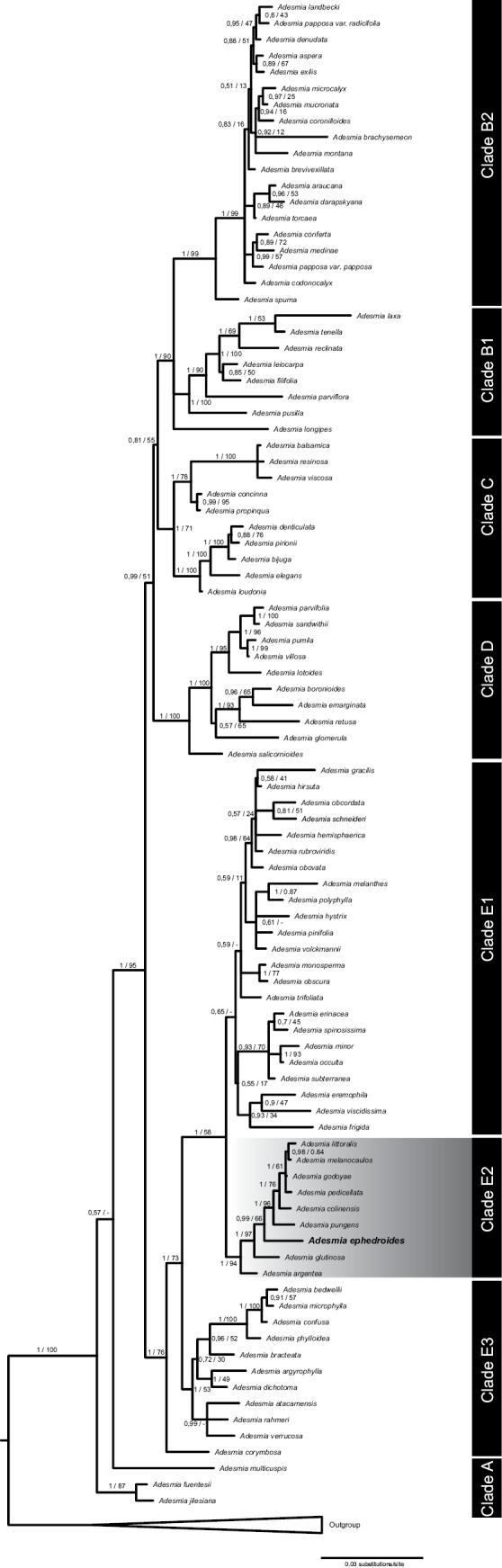
Phylogeny of *Adesmia* resulting from Bayesian Inference (BI) analyses of the combined five regions of nuclear DNA. For each node, the values of Bayesian posterior probabilities (PP) and bootstrap support (BS) under Maximum Likelihood (ML) are to the left and right of the slash, respectively. Clade names follow Pérez et al. (2024). Clade E2 is highlighted. The new species, *Adesmiaephedroides*, is highlighted in bold.

**Figure 3.**
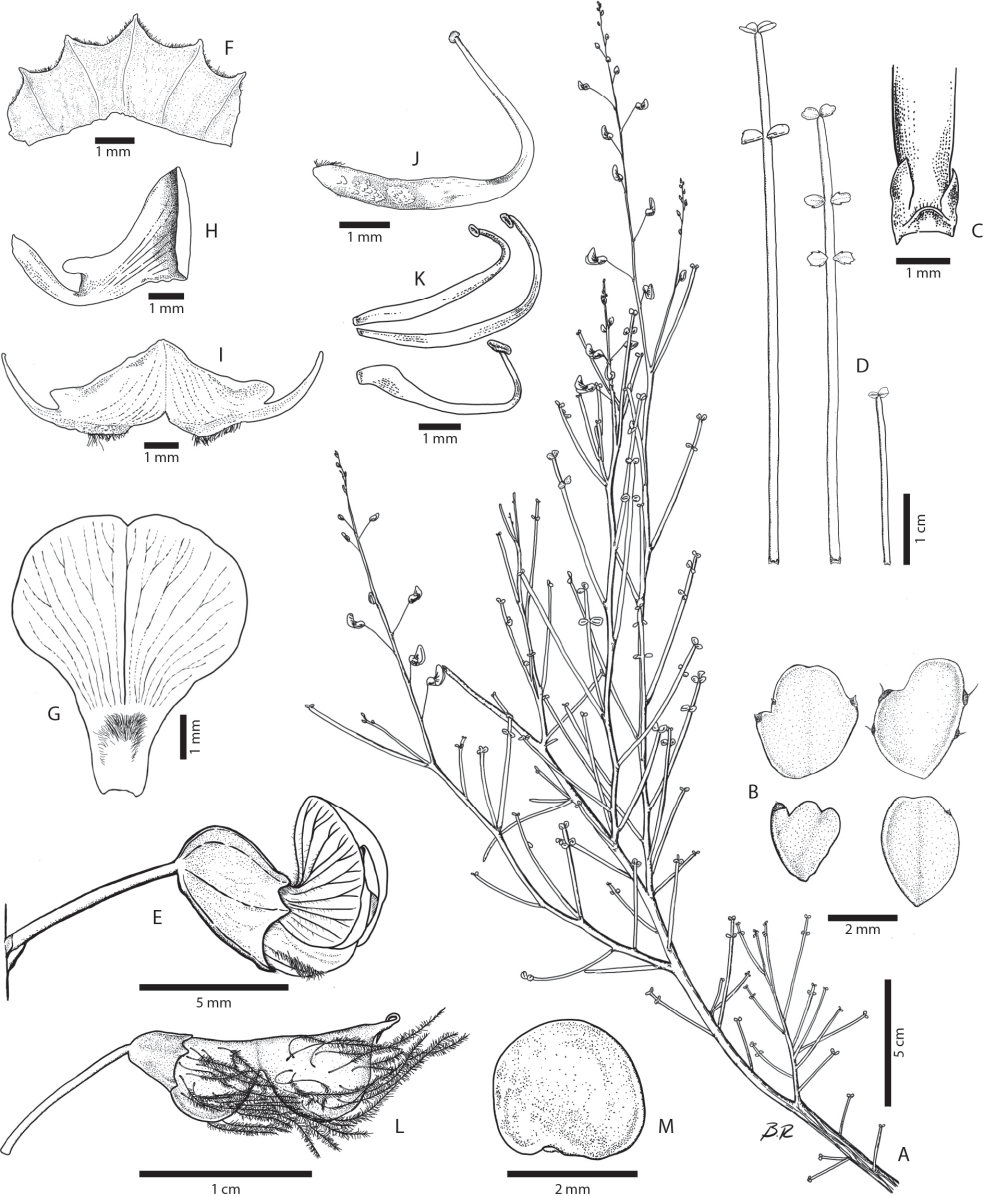
*Adesmiaephedroides* Rosende & Lavandero **A** flowering branch and leaves **B** leaflets, adaxial face **C** stipules **D** leaves, adaxial face **E** flower, side view **F** calyx, outside view **G** standard petal **H** wing petal **I** keel petals, outside view **J** gynoecium **K** stamens **L** pod **M** seed. Drawn by Benito Rosende **A–M** based on the type specimen.

**Figure 4.**
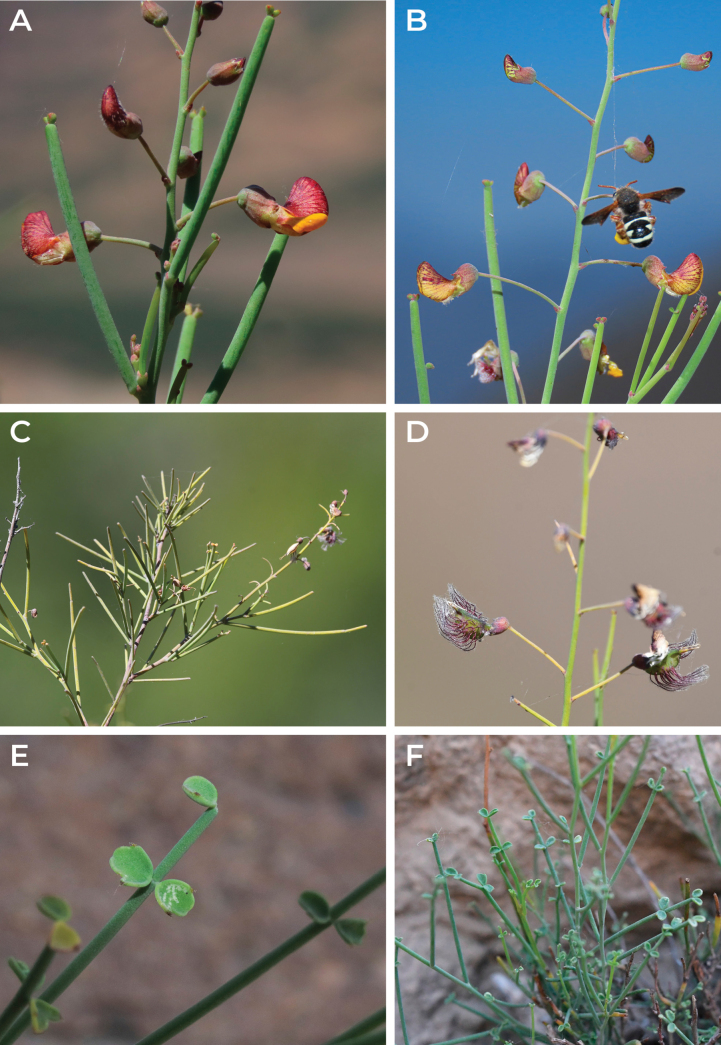
*Adesmiaephedroides* Rosende & Lavandero, sp. nov. **A** raceme, detail of flowers and leaves **B** flowering raceme with a floral visitor (*Anthidium* sp.) **C** branch with persistent leaves lacking deciduous leaflets and mature bristled fruits **D** raceme, detail of immature fruits **E** leaflets detail **F** leaves detail with fully developed leaflets. Photographed by Nicolás Lavandero (**A, B, E**) and Benito Rosende (**C, D, F**).

**Figure 5.**
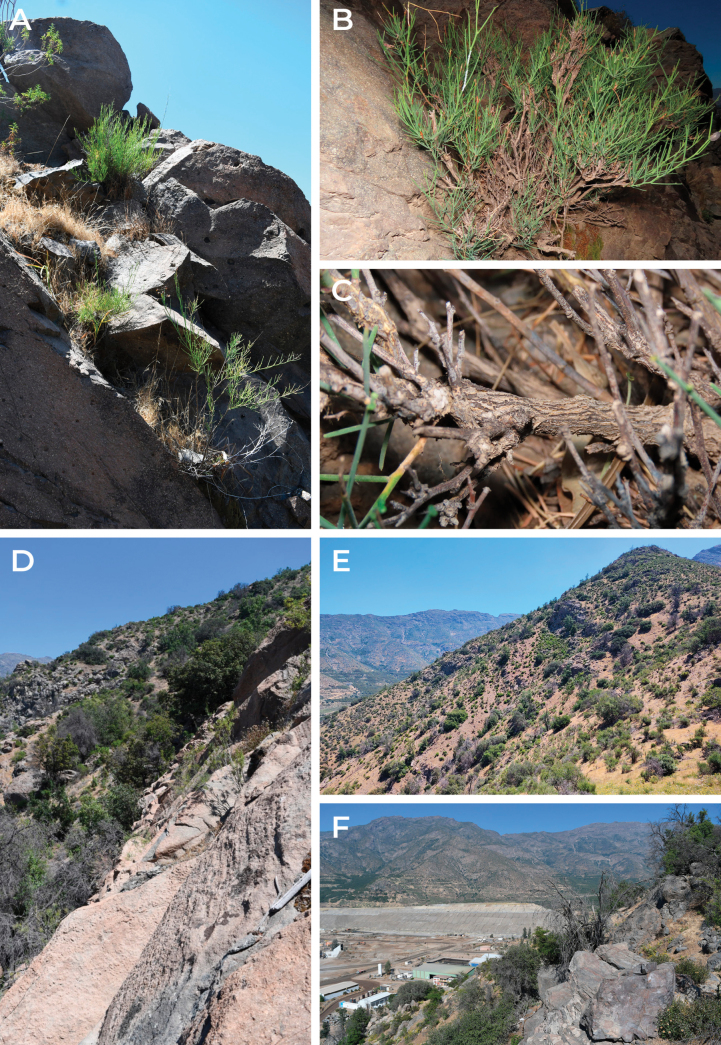
*Adesmiaephedroides* habit and habitat **A, B** habit of *A.ephedroides*, growing in rocky substrate **C** basal branch, detail of longitudinal fissured bark **D, E***A.ephedroides* habitat, rocky outcrops surrounded by sclerophyllous open forest of *Lithraeacaustica* and *Quillajasaponaria***F** mining facilities and avocado crops visible near type location. Photographed by Benito Rosende (**A, D, E, F**) and Nicolás Lavandero (**B, C**).

##### Phenology.

Flowering occurs primarily between October and December, occasionally extending until March. Fruiting is observed between November and March.

##### Etymology.

The specific epithet *ephedroides* alludes to its resemblance to the genus *Ephedra* Tourn. ex L., primarily due to its shrubby habit, colour and the arrangement of its numerous young, vertically orientated green branches. Additionally, the cylindrical leaves with deciduous leaflets contribute to an appearance reminiscent of a leafless shrub, similar to *Ephedrachilensis* Miers, with which *A.ephedroides* grows in sympatry.

##### Preliminary conservation status.

*Adesmiaephedroides* is provisionally assessed as Critically Endangered (CR) under IUCN criteria B1ab(iii,v)+B2ab(iii,v). Its extent of occurrence is estimated at 0.038 km^2^ (< 100 km^2^) and its area of occupancy is approximately 4 km^2^ (< 10 km^2^). A single population of 78 mature individuals has been recorded, despite extensive surveys in suitable habitats and seasons across surrounding areas. The species is severely threatened by land-use changes from mining and agricultural expansion. Additionally, invasive herbivores, including rabbits, goats and cattle, heavily graze on the species, impairing its growth and limiting natural regeneration. Further sampling is required to clarify the species’ distribution and population status.

### ﻿Key to the shrub and subshrub species of *Adesmia* in the coastal range of Valparaíso region, north of the Aconcagua river

This key encompasses all known shrub and subshrub species of *Adesmia* occurring in the Coastal Range of central Chile, north of the Aconcagua River. It is based on an ongoing revision of the genus, integrating field observations, herbarium specimens, phylogenetic data and systematic treatments by Burkart (1967) and Ulibarri (1986).

**Table:** 

1	Armed shrubs (with spines)	**2**
–	Unarmed shrubs (without spines)	**3**
2	Flowers arranged in racemes ending in a spiny apex	***A.microphylla***
–	Solitary flowers or small clusters arising from brachyblasts	***A.confusa***
3	Leaflet blades with conspicuous resiniferous glands, lomentaceous fruit without plumose trichomes	**4**
–	Leaflet blades without resiniferous glands, lomentaceous fruit with plumose trichomes	**5**
4	Leaflet margin entire, 6.9–11.7 mm long	***A.resinosa***
–	Leaflet margin dentate, 1.4–3.4 mm long	***A.balsamica***
5	Leaf petiole flattened (with phylloid structure)	***A.phylloidea***
–	Leaf petiole cylindrical or canaliculate	**6**
6	Leaflets lanceolate, length at least 3 times the width; flowers arranged in congested racemes; calyx teeth equal to or longer than tube	***A.rubroviridis***
–	Leaflets oblong, elliptical or obovate, length no more than twice the width; flowers arranged in elongate racemes; peduncle distinctly longer than pedicels; calyx teeth shorter than tube	**7**
7	Petiole canaliculate, indumentum sericeous and/or glandular	***A.colinensis***
–	Petiole cylindrical, indumentum glabrous or subglabrous	***A.ephedroides***

## ﻿Discussion

*Adesmiaephedroides* is characterised as an unarmed shrub with racemose inflorescences, slender, persistent leaves featuring a cylindrical petiole and rachis, a reduced number of obovate, deciduous leaflets and plumose pods (Figs 3, 4). Following Burkart’s (1967) infrageneric classification, this species would be placed in the subgenus Adesmia; however, its specific series affiliation remains unresolved. Although growth form, inflorescence structure and fruit indumentum suggest a potential affinity with Series *Argenteae* and *Bracteatae* (Burkart 1967), *A.ephedroides* exhibits key differences in leaf and stem morphology and indumentum, thus complicating its assignment to a particular series.

Phylogenetic analyses place *Adesmiaephedroides* within the E2 clade (Fig. 2) *sensu*Pérez et al. (2024), which includes species from several series described by Burkart (1967). This suggests that morphological traits such as spinescence, leaves, stem and fruit indumentum, inflorescence morphology and growth form are not consistently reliable for delineating clades within *Adesmia* (Pérez et al. 2024). Within the E2 clade, species share attributes, such as growth form (shrubs or subshrubs), flowers arranged in racemes and plumose trichomes on fruits. Moreover, they exhibit a shared geographical distribution, largely restricted to the ecotone between the southern Atacama Desert and the Mediterranean-type ecosystem of central Chile, a semi-arid region characterised by widespread shrub and xerophytic formations. Within this zone, many *Adesmia* species are a significant component of the shrub layer (Luebert and Pliscoff 2017). Coincidentally, it is the one that harbours the greatest richness and endemism of the genus within Chile (Rodríguez et al. 2018).

Persistent leaf rachis with deciduous leaflets, as observed in *Adesmiaephedroides* (Fig. 4), are uncommon in *Adesmia*. Only *Adesmiaphylloidea* has a phylloid-like flattened petiole and rachis as the primary photosynthetic structures, with small, deciduous leaflets (Burkart 1967). However, other species, such as *A.aphylla*, *A.atacamensis*, *A.bracteata* and *A.trifoliata*, possess ephemeral leaves, but maintain other photosynthetically active and persistent structures, such as bracts or stipules (Burkart 1967). These species, occurring in arid environments, likely reduce leaf area as a strategy for drought stress tolerance (De Micco and Aronne 2012). This contrasts with other shrub species from the E3 clade (*A.confusa*, *A.bedwellii*, *A.microphylla*) and E2 (*A.glutinosa* and *A.godoyae*), amongst others, which are summer deciduous species, representing a drought stress avoidance strategy (Volaire 2018).

The conservation prospects for *Adesmiaephedroides* appear dire. Based on its confinement to a single known locality, restricted area of occupancy and low number of individuals, we propose a conservation assessment of Critically Endangered (CR). Furthermore, poor regeneration has been observed at the type locality. The proximity of mining facilities and agricultural fields (within a one-kilometre radius; Fig. 1) indicates that land-use change poses a major threat to this species, exacerbated by extensive browsing damage on a significant proportion of individuals, likely caused by the invasive European rabbit (*Oryctolaguscuniculus*) as evidenced by faecal pellets at the site (pers. obs.), further underscoring its precarious situation. Although the potential for discovering additional populations in adjacent zones cannot be entirely discounted, *A.ephedroides* is not currently found within any protected areas in Chile. Despite the “El Melón” mountain formation having been included as a priority site for biological conservation by the Ministry of Environment of Chile (MMA 2025) and the documented presence of multiple species of conservation concern (Flores-Toro and Amigo 2013), it lacks protected area designation. Land-use change represents a major driver of native vegetation loss in central Chile (Schulz et al. 2010). In recent decades, the region’s sclerophyllous forests, shrublands and xerophytic vegetation cover have been significantly reduced due to the expansion of hillside agricultural areas (Duran-Llacer et al. 2025). Additional contributing threats include anthropogenic fires, overgrazing, invasive species and climate change. Central Chile is experiencing a strong trend of decreasing precipitation, as recorded in recent years (Garreaud et al. 2020).

The distinctive traits of *Adesmiaephedroides* suggest a suite of attributes associated with abiotic stress tolerance. Rupicolous plants usually show narrow distribution ranges and high endemism; the underlying evolutionary processes that explain that pattern can be due to the presence of micro-refugia, in-situ speciation, dispersal limitation and pre-adaptations to rupicolous conditions (Irl et al. 2015). A significant proportion of endemic species in montane regions are obligate rupicolous taxa (Larson et al. 2005). In Chile, rock-dwelling plant communities have been understudied (García 2010) and many recently described plant species are exclusively rupicolous with highly restricted distribution ranges (Lavandero et al. 2020; Villarroel et al. 2021; Watson et al. 2021; Santilli et al. 2022; Villarroel et al. 2022; Menegoz et al. 2024). This suggests that inaccessible areas, both in the Andes and the Coastal Mountain Range, may harbour undiscovered populations or species yet to be described. The discovery of this new species in a rocky environment underscores the floristic richness of the central coastal mountain range and the broader Chilean biodiversity hotspot, emphasising the need for increased sampling of difficult-access sites, which are not limited to high-altitude areas or vegetation-limit zones, but include, as in this case, rocky foothill zones.

## Supplementary Material

XML Treatment for Adesmia ephedroides

## ﻿Additional information

### Author ORCIDs

Benito Rosende https://orcid.org/0009-0007-7433-8118

Nicolás Lavandero https://orcid.org/0000-0002-2390-5078

Daniela Araneda https://orcid.org/0009-0001-7989-1402

María Fernanda Pérez https://orcid.org/0000-0001-5730-9760

### Data availability

All of the data that support the findings of this study (supporting this study’s findings) are available in the main text of the Suppl. material 1.

## Supplementary materials

Supplementary material 1 GenBank accession numbers used in this study Data type xlsx Explanation note List of *Adesmia* species considered in this study and respective GenBank accession numbers. Accessions in bold are new to this study. https://binary.pensoft.net/file/1371457This dataset is made available under the Open Database License (http://opendatacommons.org/licenses/odbl/1.0/). The Open Database License (ODbL) is a license agreement intended to allow users to freely share, modify, and use this Dataset while maintaining this same freedom for others, provided that the original source and author(s) are credited.Benito Rosende, Nicolás Lavandero, Daniela Araneda, María Fernanda Pérez
